# Spontaneous retroperitoneal hematoma with duodenal obstruction with diagnostic use of endoscopic ultrasound: A case series and literature review

**DOI:** 10.1007/s12328-023-01780-3

**Published:** 2023-03-24

**Authors:** Makomo Makazu, Kazuya Koizumi, Sakue Masuda, Ryuhei Jinushi, Kento Shionoya, Toshitaka Tsukiyama

**Affiliations:** 1 Gastroenterology Medicine Center, Shonan Kamakura General Hospital, 1370-1 Okamoto, Kamakura, Kanagawa 247-8533 Japan; 2 Interventional Radiology Center, Shonan Kamakura General Hospital, 1370-1 Okamoto, Kamakura, Kanagawa 247-8533 Japan

**Keywords:** Retroperitoneal hematoma, Duodenal obstruction, Aneurysm, Endoscopic ultrasound, Median arcuate ligament syndrome

## Abstract

Spontaneous retroperitoneal hematoma is rare and can cause duodenal obstruction. We report four cases of spontaneous retroperitoneal hematoma with duodenal obstruction, wherein endoscopic ultrasound was useful for diagnosis. The patients complained of vomiting with stable vital signs. Computed tomography, esophagogastroduodenoscopy, and endoscopic ultrasound findings were similar in all cases. Contrast-enhanced computed tomography revealed a low-density mass around the 2nd to 3rd part of the duodenum. Esophagogastroduodenoscopy showed an edematous, reddish, but non-neoplastic duodenal mucosa with stenosis of the lumen. Endoscopic ultrasound revealed a low-echoic mass around the duodenum and high-echoic floating matter suggesting debris and anechoic areas that indicated a liquid component. These findings suggested hematomas or abscesses. Although pseudoaneurysm of the pancreaticoduodenal artery was suspected in Case 3, we chose conservative treatment because the aneurysm was small. In Case 4, median arcuate ligament syndrome was suspected on angiography. No aneurysms or arteriovenous malformations were found; thus, endovascular embolization was not performed. The patients were treated conservatively and discharged within 3–5 weeks. English literature queries on spontaneous retroperitoneal hematoma with duodenal obstruction in MEDLINE revealed 21 cases in 18 studies. The clinical features of these patients and the present four cases have been discussed.

## Introduction

Spontaneous retroperitoneal hematoma (SRH) is rare but potentially fatal clinically, defined as bleeding into the retroperitoneal space without trauma or iatrogenic manipulation. Most literature on SRH is limited to case series and retrospective cohort studies. Abdominal pain was found to be the most common symptom [1]. Other symptoms include pain in the back, flank, and hips. Symptoms of hypovolemia, such as syncope, pallor, and dizziness, have further been reported [2]. Although SRH sometimes causes duodenal obstruction (DO) and leads to vomiting, reports of such symptoms are rare [3].

SRH is mainly diagnosed using contrast-enhanced computed tomography (CECT). SRH with DO sometimes resembles malignant tumors of the pancreas, duodenum, and surrounding tissue [4]. Endoscopic ultrasound (EUS) has been used to observe and collect samples from the pancreas, gastrointestinal (GI) tract, posterior mediastinum, and retroperitoneum [5]. However, previous English literature that used EUS for SRH diagnosis was not found. Here, we present four cases of SRH with DO using EUS for the diagnosis.

## Case reports

### Case 1

**Fig. 1:**
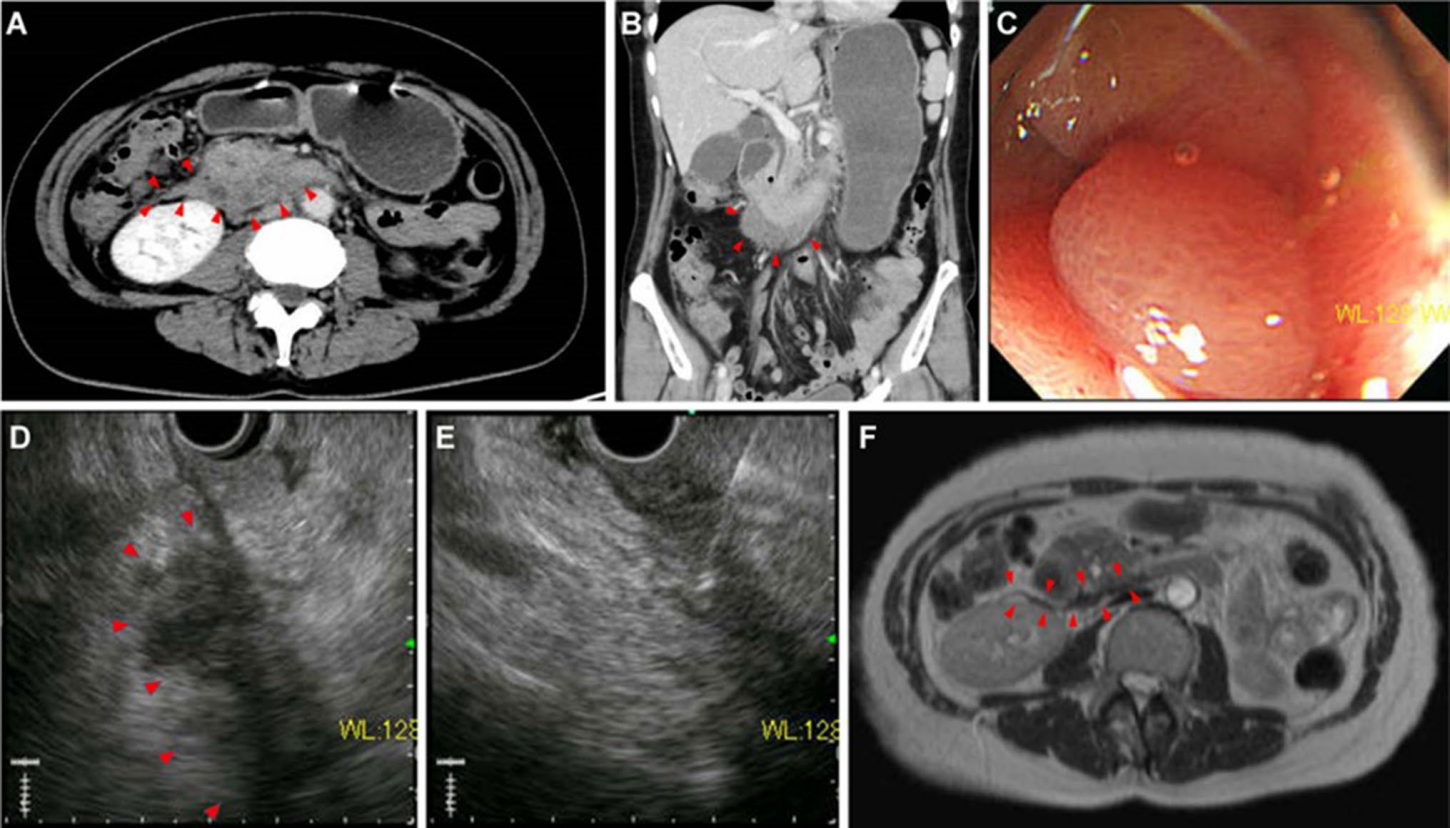
A 51-year-old female presenting with epigastric pain and vomiting (Case 1). **a** A low-density, band-shaped area spreading from the paraduodenal space to the right anterior pararenal extraperitoneal space was identified (arrowheads). The walls of the 2nd and 3rd part of the duodenum were thickened and in contact with the low-density area. **b** In coronal-reformatted images, the low-density area was situated on the caudal side of the 2nd to 3rd part of the duodenum and lead to its obstruction (arrowheads). **c** The esophagogastroduodenoscopy (EGD) on day 1 showed edematous, erythematous duodenal mucosa and narrowing of the lumen to the inferior duodenal angle. The endoscope did not pass through the lumen. **d** Endoscopic ultrasound (EUS) on day 5 revealed a band-shaped low echoic area just behind the 2nd part of the duodenum (arrowheads). **e** Transduodenal EUS-guided fine-needle aspiration/biopsy was performed to assess the possibility of a malignant lesion. **f** T2-weighted image of MRI on day 17 revealed no pancreatic tumor and no abnormal signal in the duodenal wall (arrowheads)

A 51-year-old female presented to our emergency department with a 3-day history of epigastric pain and vomiting. The patient’s vital signs were normal. Physical examination revealed tenderness of the epigastric region. Laboratory tests showed a white blood cell count of 7,600/µL, C-reactive protein (CRP) of 2.186 mg/dL, and D-dimer of 6.9 µg/mL. She did not have a history of anticoagulant medication or abdominal trauma. Abdominal CECT on day 1 revealed a markedly distended stomach and proximal duodenum. There was a low-density, band-shaped area spreading from the paraduodenal space to the right anterior pararenal extraperitoneal space (Fig. 1a, b). The walls of the 2nd and 3rd part of the duodenum were thickened and were in contact with the low-density area. There were no apparent findings of aneurysms or extravasation of contrast medium around the mass. Esophagogastroduodenoscopy (EGD) on day 1 showed an edematous, erythematous duodenal mucosa and narrowing of the lumen of the inferior duodenal angle (Fig. 1c). The endoscope was not passed through the lumen. The patient was managed using nasogastric tube suction. On day 3, dynamic CECT was performed because pancreatic cancer was suspected. No apparent pancreatic tumor or remarkable changes were noted in the low-density area. The patient underwent EUS on day 5. A band-shaped, low-echoic area behind the 2nd part of the duodenum (Fig. 1d) was accompanied by a partially anechoic component and a high-echoic component. EUS-guided fine-needle aspiration biopsy (EUS-FNAB) was performed to assess the possibility of a malignant lesion transduodenally (Fig. 1e). The pathological diagnosis was the presence of a clot, with no malignant findings; therefore, the patient was diagnosed with SRH. EGD on day 11 revealed a slight improvement in the extrinsic compression of the duodenum. On that day, the nasogastric tube was removed, and the patient started drinking water. She gradually returned to a normal diet by day 15. Abdominal magnetic resonance imaging (MRI) on day 17 revealed no pancreatic tumor or abnormal signal in the duodenal wall (Fig. 1f). The region that was imaged as a low-density area on CT showed low intensity on both T1- and T2-weighted images. The patient was discharged on day 19. CECT performed 2 months later showed the disappearance of almost all hematomas. We consulted a radiologist and decided not to perform angiography because there was no evidence of abdominal visceral aneurysms on the previous CT scans. She had not undergone CT since then. Six years later, we called her about her condition, and she said she had no symptoms.

### Case 2

**Fig. 2:**
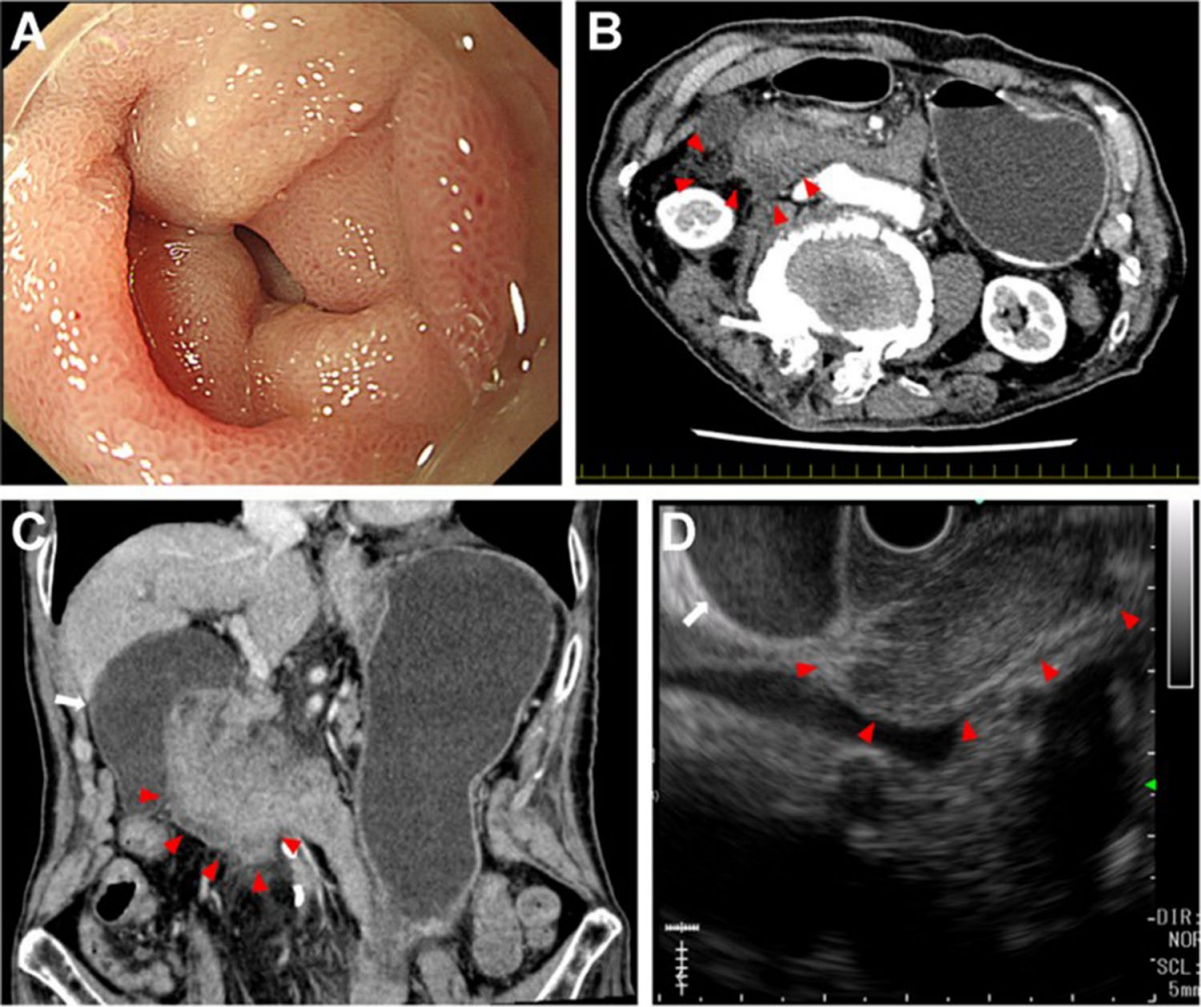
An 83- year-old male presenting with fever and vomiting (Case 2). **a** The EGD on day 3 showed edematous, reddish mucosa and narrowed lumen of the 2nd part of the duodenum. The endoscope did not pass through the lumen. **b** A dynamic contrast-enhanced CT on day 4 revealed a band-shaped low-density area spreading from the paraduodenal space to the right anterior pararenal extraperitoneal space (arrowheads) and appears to be in contact with the thickened wall of the 2nd and 3rd part of the duodenum. **c** In coronal reformatted images, the low-density area is located on the caudal side of the 2nd to 3rd part of the duodenum and obstructs it (arrowheads). There was a distention of the stomach and an enlargement of the gallbladder (white arrow). **d** EUS on day 11 revealed a 36-mm-sized oval-shaped low echoic mass (arrowheads) just outside of the thickened wall of the 2nd part of the duodenum. It extended along with the 3rd part of the duodenum in an L shape. A small amount of anechoic component and partially high echoic component were observed in the low echoic mass. An enlargement of the gall bladder (white arrow) was further identified

An 83-year-old male presented to our emergency department with a 1-day history of fever and vomiting. His temperature had risen to 37.5 °C; however, his other vital signs were stable. He was bedridden because of left hemiplegia caused by an infarction in the right cerebral hemisphere. He usually took aspirin but discontinued it after hospitalization. The patient had no history of trauma. Physical examination revealed no abdominal pain or tenderness. Laboratory studies showed a white blood count of 11,600/μL, Hb of 9.9 g/dL, and CRP of 6.138 mg/dL. Computed tomography of the head revealed no acute cerebral infarction. On day 2, abdominal ultrasonography (AUS) revealed enlargement of the gallbladder. EGD on day 3 showed edematous, erythematous mucosa and narrowing of the lumen of the 2nd part of the duodenum (Fig. 2a). The endoscope was not passed through the lumen. The patient underwent abdominal dynamic CECT on day 4 for suspected pancreatic cancer. There was a band-shaped low-density area spreading from paraduodenal space to the right anterior pararenal extraperitoneal space and in contact with the thickened walls of the 2nd and 3rd part of the duodenum (Fig. 2b, c). No apparent findings of aneurysms or extravasation of the contrast medium were noted around the mass. On day 5, the patient again underwent AUS. This revealed the thickening of the 2nd part of the duodenum and the low-echoic area around it. Based on these findings, retroperitoneal hematoma was suspected rather than pancreatic cancer. The patient’s condition was managed using nasogastric tube suction. On day 11, EUS was performed. There was a 36-mm-sized oval shaped, low-echoic mass just outside the 2nd part of the duodenum. It extended along the 3rd part of the duodenum in an L shape (Fig. 2d). A small number of anechoic and partially high-echoic components were observed in the low echoic mass. The presence of a retroperitoneal hematoma was suspected based on these findings. We planned to monitor the patient conservatively and consider EUS-FNAB if the mass did not shrink. Abdominal CECT on day 18 showed an improvement in the wall thickness of the 2nd to 3rd part of the duodenum. The band-shaped low-density area slightly decreased. Retroperitoneal hematoma was diagnosed on the basis of the shrinking trend of the mass. He started drinking water on day 21, and he was put on a liquid diet on day 25 and a normal diet on day 27. He was readministered aspirin on day 26 and discharged on day 33. No recurrence of SRH was observed until the patient died of aspiration pneumonia 4 years later.

### Case 3

**Fig. 3:**
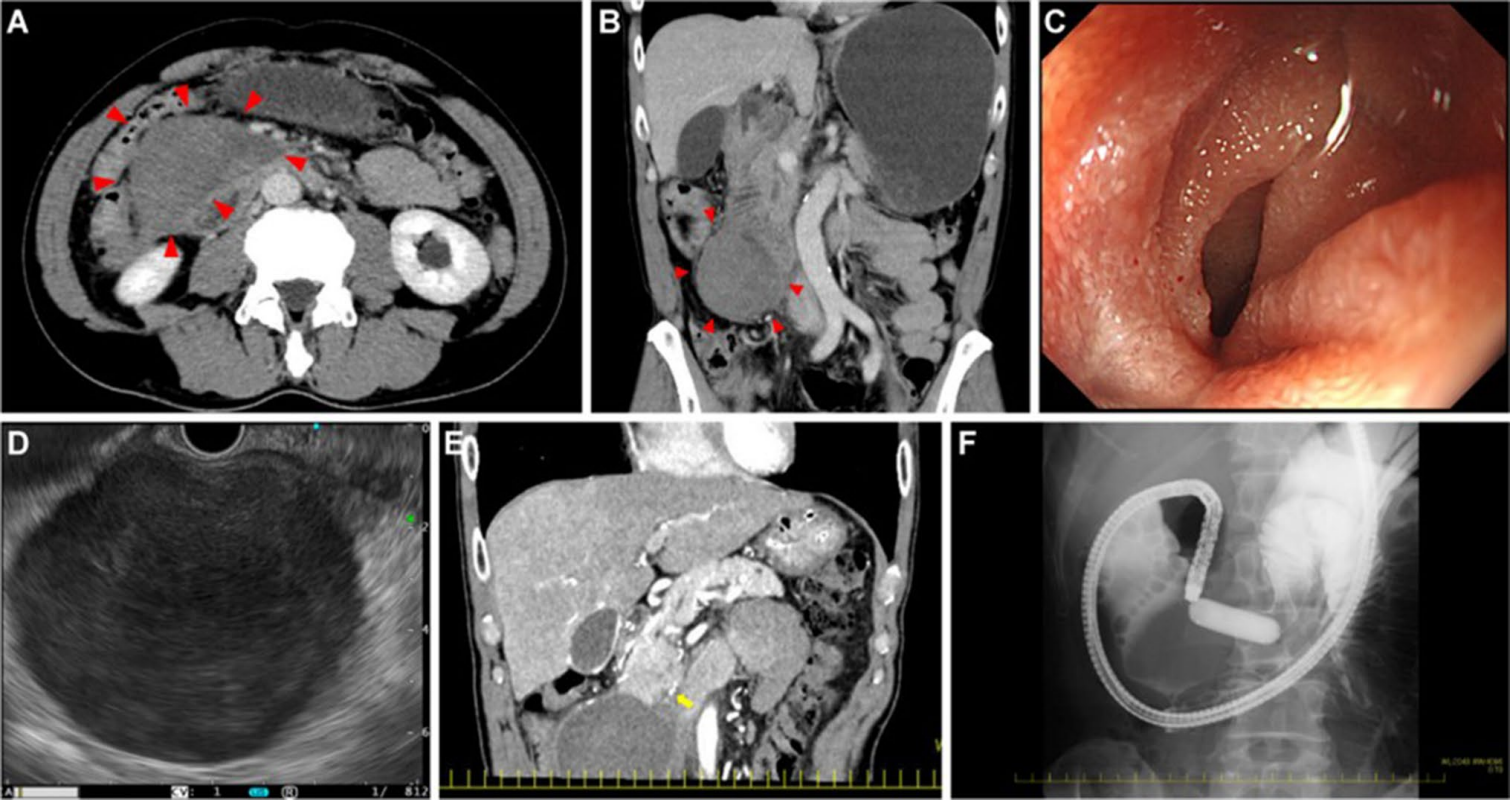
A 62-year-old male presenting with epigastric pain and vomiting. **a** Abdominal contrast-enhanced CT on day 1 revealed a low-density, 6-cm-sized mass (arrowheads) at the retroperitoneal space around the 2nd to 3rd part of the duodenum. **b** In coronal reformatted images, the inferior duodenal angle to the 3rd part of the duodenum was compressed due to the mass (arrowheads) and caused the distention of the stomach and 1st to 2nd part of the duodenum. **c** EGD on day 2 revealed stenosis of the 3rd part of the duodenum. Although the upper endoscope did not pass through the stenosis due to its bending in the stomach, a short-type, balloon-assisted endoscope could pass through there. No neoplastic lesion was identified on the mucosal surface. **d** EUS was performed after the EGD. A 7-cm-sized mass with a clear margin was observed just outside of the 2nd part of the duodenum The mass showed low echogenicity with a partially anechoic area and a debris-like area of slightly high echogenicity. **e** Contrast-enhanced computed tomography on day 14 revealed no change in the size of the hematoma. A pseudoaneurysm of the posterior inferior pancreaticoduodenal artery (yellow arrow) was suspected in the left edge of the hematoma. This image was previously used in a Japanese case report ([6]). **f** EGD on day 23 still revealed duodenal stenosis. The patient underwent endoscopic balloon dilation on the stenosis

This case has been published in a Japanese journal [6]. A 62-year-old male presented to our emergency department with a 7-day history of epigastric pain and vomiting. The patient’s vital signs were normal. Physical examination revealed tenderness of the epigastric region. Laboratory studies revealed no anemia or coagulation disorders. He did not have a history of anticoagulant medications or abdominal trauma. Abdominal CECT on day 1 revealed a low-density, 6-cm-sized mass at the retroperitoneal space around the 2nd to 3rd part of the duodenum (Fig. 3a, b). The 3rd part of the duodenum was compressed due to the mass, causing distention of the stomach and 1st to 2nd part of the duodenum. No apparent aneurysms or extravasation of contrast medium around the mass were observed. EGD on day 2 revealed stenosis in the 3rd part of the duodenum (Fig. 3c). Although the upper endoscope did not pass through the stenosis due to the bending of the scope in the stomach, a short-type balloon-assisted endoscope could pass through it. Neoplastic lesions were not observed on the mucosal surface. EUS was performed after the EGD. A 7-cm-sized mass with a clear margin just outside the 2nd part of the duodenum was described (Fig. 3d). The mass showed low echogenicity, with a partial anechoic area and a debris-like slightly high-echoic area. Retroperitoneal hematoma was diagnosed based on these imaging findings. The patient was managed using nasogastric tube suction. CECT on day 14 revealed no change in hematoma size. A pseudoaneurysm of the posterior inferior pancreaticoduodenal artery was suspected at the left edge of the hematoma (Fig. 3e). Angiography was not performed because the pseudoaneurysm was very small and the hematoma was not enlarged. Although abdominal plain CT on day 21 showed a slightly diminishing hematoma, EGD on day 23 still revealed duodenal stenosis. The patient underwent endoscopic balloon dilation for stenosis (Fig. 3f). The nasogastric tube was subsequently removed. He started on a liquid diet on day 28 and gradually returned to a normal diet. The patient was discharged on day 34. CECT performed 4 months later revealed a markedly decreased hematoma. There were no apparent pseudoaneurysms. Another 5 months later, CECT showed hematoma shrinkage of 14 mm in size. There were no apparent findings of aneurysm. We consulted a radiologist and decided not to perform the angiography. We called the patient about his condition 2 years later, and he said he had no symptoms.

### Case 4

**Fig. 4:**
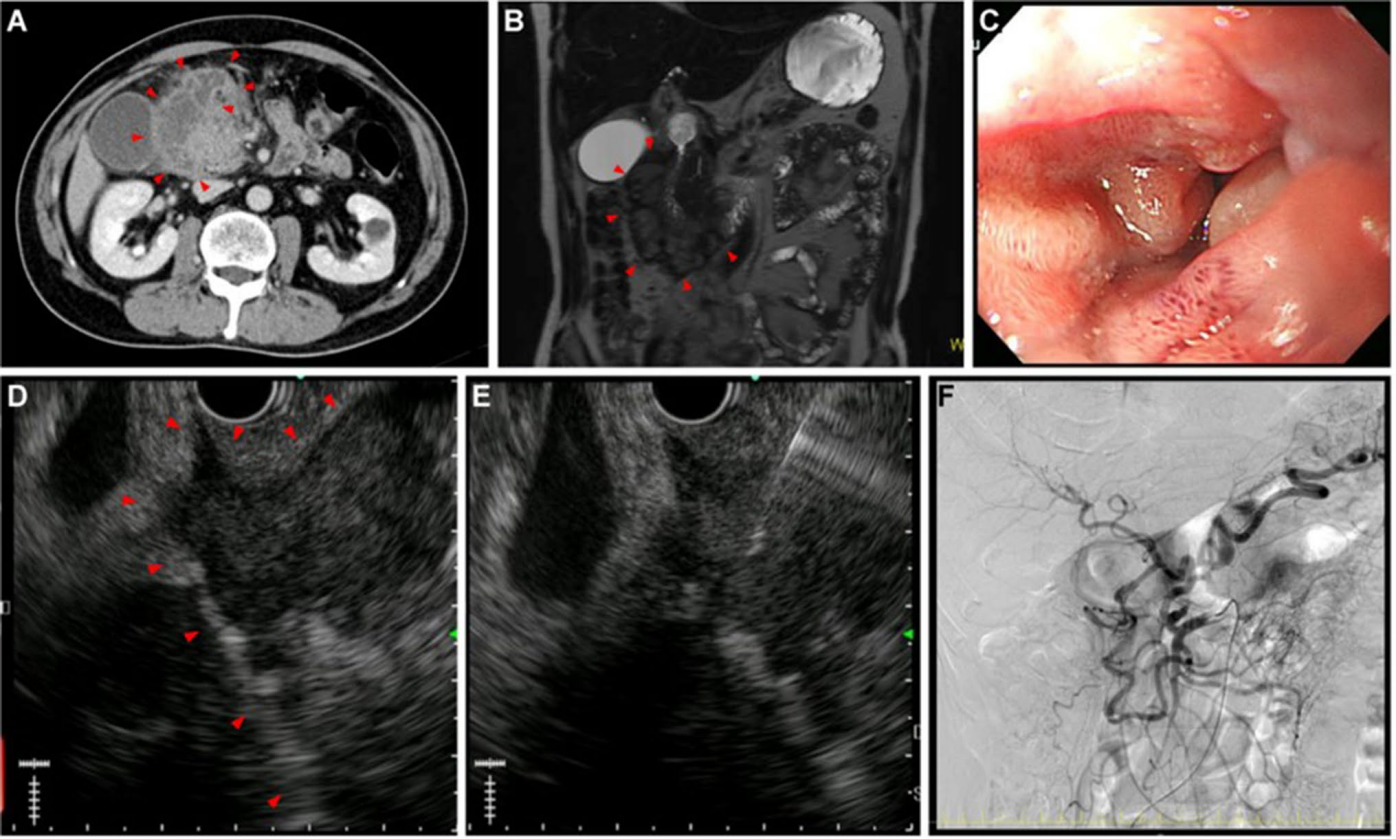
A 64-year-old male presenting with abdominal fullness and vomiting. **a** Abdominal contrast-enhanced CT on day 2 revealed a 9-cm-sized, low-density mass (arrowheads) around the 2nd to 3rd part of the duodenum. The capsule and internal septum of the mass were enhanced. **b** Abdominal contrast-enhanced magnetic resonance imaging on day 3. On T2-weighted image, the rim of the mass each revealed a weak signal and the inside of the mass revealed a middle signal (arrowheads). **c** EGD on day 6 showed edematous stenosis of the 2nd portion of the duodenum. **d** EUS on day 9 revealed a large cystic mass around the duodenum (arrowheads). The mass showed low echogenicity with a partially anechoic area and a debris-like area of slightly high echogenicity, and these were not obvious findings of a malignant tumor. There was no apparent vessel structure in the mass. **e** Fine-needle biopsy was performed to assess the possibility of infection. The pathological result was the existence of peripheral blood with a small amount of fibrous tissue and glandular epithelium. No bacteria were identified in the culture test. **f** An angiography on day 15. Selective angiography of the superior mesenteric artery (SMA) showed a remarkably dense network of collateral vessels connecting the SMA to the celiac artery. There was an irregular caliber change in the anterior pancreaticoduodenal arcade. Since there were no apparent findings of aneurysms or arteriovenous malformation, endovascular embolization was not performed

A 64-year-old male presented to our emergency department with a 4-day history of abdominal fullness. Laboratory studies showed mild elevations in white blood cell count and CRP levels. The AUS showed no remarkable findings. The patient was discharged with antibiotic treatment. He presented a second time after 9 days with persistent abdominal fullness and vomiting from the previous day. His heart rate was elevated to 105/min; his other vital signs remained stable. Physical examination revealed a palpable mass with mild tenderness in the upper right quadrant. Laboratory studies showed a white blood cell count of 13,600/μL and CRP of 7.61 mg/dL. No anemia was noted. He did not have a history of anticoagulant medications or abdominal trauma. Abdominal plain CT on day 1 revealed a 9-cm low-density mass around the 2nd to 3rd part of the duodenum. CECT on day 2 revealed an enhanced capsule and internal septum of the mass (Fig. 4a). There were no apparent findings of aneurysms or extravasation of contrast medium around the mass. The patient was started on a liquid diet on day 2. Abdominal contrast-enhanced MRI on day 3 showed a weak, high signal in the mass on T1-weighted images. On T2-weighted images, the rim of the mass revealed a weak signal and its interior displayed intermediate intensity (Fig. 4b). Diffusion-weighted images showed a strong high signal with a decreased apparent diffusion coefficient. The contrast medium enhanced the rim and internal septum of the mass. EGD on day 6 showed edematous stenosis in the 2nd portion of the duodenum (Fig. 4c). No neoplastic findings were present in the mucosa, and the endoscope could pass through the lumen. The patient underwent EUS on day 9 when a large cystic mass was observed around the duodenum (Fig. 4d) that showed low echogenicity with a partial anechoic area and a debris-like high echoic area. No apparent vessel structures were observed in the mass. Although the mass was not suspected to be malignant, EUS-FNAB was performed because infection of the hematoma could not be ruled out clinically due to the high level of inflammatory reaction and irregular shape of the mass (Fig. 4e). The fluid was highly viscous; therefore, only a small amount of blood could be aspirated which included no obvious pus. The pathological result was the presence of peripheral blood, with small amounts of fibrous tissue and glandular epithelium, and no bacteria identified in the cultures. Based on imaging findings and pathology, we diagnosed the mass as a retroperitoneal hematoma without infection. CECT on day 12 showed a remarkably diminished mass. Stenosis of the celiac trunk due to median arcuate ligament syndrome (MALS) was suspected on CT. On day 15, the patient underwent angiography to examine the presence of any abdominal visceral aneurysms associated with MALS (Fig. 4f). Selective catheter injection into the celiac artery was impossible due to severe stenosis of the origin. Selective angiography of the superior mesenteric artery (SMA) revealed a remarkably dense network of collateral vessels connecting the SMA to the celiac artery. We identified the presence of a slightly irregular caliber change in the anterior pancreaticoduodenal arcade. As there were no apparent findings of aneurysms or arteriovenous malformations, endovascular embolization was not performed. On day 13, the patient was fed a normal diet and was discharged on day 17. CECT performed 2 months later showed that the hematoma had disappeared; he had not undergone CT since then. We called him about his condition 7 years later, and he said that he had no symptoms.

The clinical characteristics and imaging features of the four cases are summarized in Table 1.

**Table 1:** Clinical characteristics and features of images of present four cases

Case number (sex/age)	1 (F/51)	2 (M/83)	3 (M/62)	4 (M/64)
Symptoms	Epigastric pain, vomiting	Fever, vomiting	Epigastric pain, vomiting	Abdominal fullness, vomiting
Vital signs	Stable	Stable	Stable	Stable
CT findings				
Location	2nd–3rd part of the duodenum to the right anterior pararenal extraperitoneal space	2nd–3rd part of the duodenum to the right anterior pararenal extraperitoneal space	2nd part of the duodenum	2nd part of the duodenum
Density	Low	Low	Low to high	Low to high
EGD findings				
Site of stenosis	2nd part of the duodenum	2nd part of the duodenum	3rd part of the duodenum	2nd part of the duodenum
Mucosal findings	Reddish, edematous but not neoplastic	Reddish, edematous but not neoplastic	Reddish, edematous but not neoplastic	Reddish, edematous but not neoplastic
Scope passage	No	No	Yes	Yes
EUS date (from being hospitalized)	5	11	2	9
EUS findings				
Surface	Irregular	Irregular	Irregular	Irregular
Fluid-like aspect	( +)	( +)	( +)	( +)
Sedimentation	( +)	( +)	( +)	( +)
Solid-like component	( +)	(−)	(−)	(−)
High-echoic component	( +)	( +)	( +)	( +)
Pathology (FNAB)	Clot	NE	NE	Peripheral blood with fibrous tissue and glandular epithelium
Aneurysm ( +) or (−)	(−)	(−)	PDAA was suspected on CT	(−) Slightly irregular caliber change in the anterior pancreaticoduodenal arcade
Treatment	Conservative	Conservative	Endoscopic balloon dilation	Conservative
Inpatient days	19	33	34	17
Outcome	CT 2 months later showed disappearance of the hematoma	Doing well but died four years later due to aspiration pneumonia	CT 9 months later showed shrunken hematoma	CT 2 months later showed disappearance of the hematoma

*CT* computed tomography, *EUS* endoscopic ultrasound, *PDAA* pancreaticoduodenal artery aneurysm, *FNAB* fine-needle aspiration/biopsy, *NE* not evaluated

## Discussion

SRH is a rare clinical entity that can lead to DO. The clinical presentation of this condition remains unclear owing to its rarity. We reviewed the English literature in MEDLINE from 1961 to 2022 for reports on SRH with DO. The terms we searched included retroperitoneal, duodenum, duodenal, obstruction, hemorrhage, hematoma, and aneurysm. We identified 21 cases in 18 literature sources [4, 7–20]. The clinical features of those 21 cases and the present four cases are described in Table 2.

**Table 2:** Twenty-five cases of spontaneous retroperitoneal hematoma with duodenal obstruction (including the present four cases)

Parameters	
Age	58 (34–83)
Sex (M/F)	16/9
Symptoms	
Vomiting	19 (76%)
Abdominal pain	16 (64%)
Nausea	6 (24%)
Abdominal fullness	5 (20%)
Bloody stool	2 (8%)
Back pain	1 (4%)
Vital signs	
Stable	14 (56%)
Unstable	1 (4%)
Not described	10 (40%)
Causes of RH formation	
Aneurysms (including suspected cases)	17 (68%)
PDAA (CA stenosis ±)	15 (8/7)
GDAA	1
Arc of buhler with CA stenosis	1
Spontaneous	8 (32%)
Anticoagulant use	1
SSRI and NSAIDs use	1
No particular cause	6
Modality used for diagnosis	
Computed tomography	25 (100%)
Esophagogastroduodenoscopy	20 (80%)
Angiography	10 (40%)
Abdominal ultrasound	8 (32%)
Magnetic resonance imaging	6 (24%)
Endoscopic ultrasound	4 (16%)
Scintigraphy	1 (4%)
Treatment	
Surgery	6 (24%)
Endovascular treatment	5 (20%)
Endoscopic balloon dilation	1 (4%)
Conservative (including nasogastric tube)	12 (48%)
Not described	1 (4%)
Clinical course	
Survival	23 (92%)
Death due to other cause	1 (4%)
Not described	1 (4%)
Inpatient days (only 10 cases described)	30.5 (13–35)

*PDAA* pancreaticoduodenal artery aneurysm, *CA* celiac artery, *GDAA* gastroduodenal artery, *SSRI* selective serotonin reuptake inhibitor, *NSAIDs* non-steroidal anti-inflammatory drugs

Regarding the symptoms of SRH with DO, SRH mainly causes abdominal, back, or flank pain and sometimes leads to unstable vital signs [1, 2]. However, among the 25 cases of SRH with DO, the most common symptom was vomiting in 76%, followed by abdominal pain in 64% and nausea in 24%. Vital signs were unstable in only one case. The other cases presented stable vital signs or lacked a description. These clinical features differ from those of SRH in patients without DO. A possible reason for this difference is the anatomical features of the duodenum and the retroperitoneum. The 2nd to 3rd part of the duodenum is surrounded by several organs and tissues, such as the pancreas, right kidney, inferior vena cava, aorta, and mesocolon. Thus, the duodenum exists in a semi-closed compartment. Therefore, when bleeding occurs in this compartment, it may remain in the early phase and cause tamponade. Although it may keep the vital signs of the patient stable, it results in duodenal compression. Increased pressure around the duodenum may secondarily cause congestive edematous thickening of the duodenal wall. Ishiyama et al. proposed the concept of duodenal compartment syndrome [3].

Regarding the diagnosis of SRH associated with DO, CT was performed in all cases. We described the features of images of the present four cases in Table 1. Typical CT findings are an irregularly shaped mass around the 2nd to 3rd part of the duodenum and distention of the stomach and proximal duodenum. Because of stable vital signs and no suspicious triggers for hematoma, such as trauma or iatrogenic procedures, it is often difficult to include SRH in the differential diagnosis. Malignant tumors of the pancreas, duodenum, and surrounding tissues are crucial differential diagnoses.

EUS can be used to observe these lesions in detail. Even if the endoscope cannot pass through the narrowed lumen, EUS can be performed on the oral side of the stenosis. To the best of our knowledge, no English literature has used EUS for the diagnosis of SRH. EUS has the following two advantages. First, EUS has a high spatial resolution, and, furthermore, it is possible to observe SRH around the duodenum from a short distance, which is difficult to observe with percutaneous AUS. Second, EUS-FNAB allows the pathological diagnosis to rule out infection or malignancy.

Regarding the first point, there were several reports about the usefulness of percutaneous AUS for the diagnosis of hematomas. Ritchie et al. have reported the findings of AUS, suggesting hematoma as an inhomogeneous pattern with variable amounts of internal echoes [21]. Badea et al. have described a case of retroperitoneal and subcapsular liver hematoma [22]. They reported that the ultrasonographic appearances of recent hematomas are fluid-like and transonic and that there can be sedimentation inside or even intense echoes generated by the blood clots. Unfortunately, peri-duodenal hematomas are often difficult to observe with percutaneous AUS. EUS can easily observe those lesions from close range without the influence of gases in the digestive tract. The spatial resolution of EUS is higher than CT or MRI. Several reports have shown that EUS is superior to CT and MRI in detecting small pancreatic cancer [23]. EUS was performed in the present four cases, and in all cases the lesions could be observed in detail. High-echoic floating matter suggesting debris and anechoic areas suggesting a liquid component were observed in all four cases. These findings increase the likelihood of the presence of hematomas or abscesses rather than malignant tumors. Although there was a report on the efficacy of contrast-enhanced EUS for the diagnosis of liver hematoma [22], this was not performed in our cases because perflubutane is covered by Japanese health insurance only for liver and breast tumors.

Regarding the second advantage, we performed EUS-FNAB in two of the four cases to help determine the treatment strategy. However, the safety of this approach is controversial. We believe that EUS-FNAB should be considered only when the abscess is suspected or malignant tumor cannot be ruled out.

As for the differential diagnosis of hematoma from the abscess, we reviewed the English literature in MEDLINE for reports on retroperitoneal hematoma with infection and found several case reports [24–26]. In all these cases, the patients presented with fever or severe inflammatory findings in the laboratory. As in these cases, if there are symptoms or laboratory findings suggestive of bacteremia, infection cannot be ruled out. In such cases, we consider EUS-FNAB to be useful.

As for the possibility of pancreatic cancer, we reviewed the English literature in MEDLINE for reports on retroperitoneal hematoma mimicking pancreatic cancer and found a report [4]. As in this case, if there is a solid component in the lesion, the possibility of cancer cannot be denied. In this case, a percutaneous needle biopsy was performed to rule out malignancy, and the patient was treated conservatively and had a favorable outcome. In addition, of the 21 cases reviewed in the present report except for our 4 cases, surgery was performed in 6 cases. In 3 of these, surgery was chosen because the possibility of a retroperitoneal tumor or pancreatic cancer could not be ruled out [7, 14, 17]. A retroperitoneal hematoma may require several weeks to shrink. If the mass-like lesion is a malignant tumor, it may increase during the waiting period. Therefore, determining whether or not the tumor is malignant is critical to the patient's prognosis. We believe that EUS-FNAB is a useful option to obtain a histological diagnosis without excessive invasion.

Regarding the treatment content, of the 14 patients that had or were suspected to have aneurysms, only 1 patient had a gastroduodenal artery aneurysm (GDAA) and the others had pancreaticoduodenal artery aneurysms (PDAAs). Stenosis of the celiac trunk was observed in 6 of the 13 PDAA cases. PDAA is reported to account for only 2% of abdominal visceral artery aneurysms [27, 28]. PDAA is divided into pseudoaneurysms and true aneurysms. The former occurs more often than the latter and is associated with inflammation such as pancreatitis and trauma [29]. The latter is very rare and associated with stenosis of the celiac trunk, arteriosclerosis, infection, and fibrodysplasia. A previous review of true PDAAs reported that 63% were associated with celiac trunk lesion [30]. The causes of celiac trunk stenosis have been reported to be median arcuate ligament syndrome (MALS), arteriosclerosis, and congenital changes. MALS occurs due to extraluminal compression of the celiac trunk by the median arcuate ligament [31]. The mechanism of aneurysm formation in MALS is as follows. Decreased blood flow in the celiac artery secondarily leads to increased blood flow from the SMA through the pancreatic arcade, which results in high internal pressure on the PDA and the formation of aneurysms.

As PDAA have a higher risk of rupture regardless of their morphology and size, proactive treatment is recommended [32]. The treatment options have changed from surgery to endovascular treatment (EVT). The latter has fewer associated complications and better outcomes than the former [33]. As for celiac trunk stenosis, it remains controversial whether stenosis should be treated in addition to aneurysm therapy [32]. Only 9 patients underwent angiography among 17 patients that were suspected to have PDAA or GDAA visceral artery aneurysm on CT underwent angiography. Of these, only five patients underwent coil embolization. Of the remaining eight patients that were suspected to have aneurysms, four underwent surgery for retroperitoneal hematoma, one underwent endoscopic balloon dilation, and the others were treated conservatively, including suction of the gastric fluid by a nasogastric tube. Although no long-term clinical courses were described for patients in the previous reports, our four patients revealed no recurrence of retroperitoneal hematoma or aneurysm on CT performed several months later. Additionally, the literature lacked a description of treatment for that for celiac trunk stenosis. In Case 4, MALS was suspected on angiography. There were no apparent findings of aneurysms; hence, EVT or treatment for MALS was not performed. Careful follow-up is required if conservative treatment is selected.

The causes of hematoma formation were unclear in 8 of the 25 cases. Of these, two underwent surgery, and the remaining six were treated conservatively. Of the 25 cases, a total of 6 underwent surgery, 5 underwent EVT, 1 case in the present report underwent endoscopic balloon dilation, 1 was not described as to its outcome, and the remaining 12 were treated conservatively. Even in those conservatively treated cases, duodenal obstruction due to retroperitoneal hematoma was relieved. Only one patient died 8 days after EVT due to cardiac arrest [10]. Although the length of hospital stay was not described in several literatures, the median number of that in 10 cases that described that information was 30.5 days (range, 13–35 days). We performed endoscopic balloon dilation in Case 3 on day 23. This procedure may be useful for the treatment of duodenal stenosis associated with fibrosis during hematoma resorption.

We reported four cases of SRH with DO wherein EUS was useful for diagnosis. We reviewed previously reported cases and our own. The clinical features of SRH with DO are different from those of previously reported retroperitoneal hematomas without DO; hence, diagnosis may be difficult. We should list this condition as one of the differential diagnoses in patients who visited the hospital with complaints of vomiting and revealed a low-density mass around the duodenum on CT. EUS may be useful for the diagnosis of SRH with DO and may help avoid unnecessary surgery. If there are no apparent findings of vessels or aneurysms around the tumor, EUS-FNAB may also be useful for ruling out malignant tumors and abscesses.
